# Reorganization of theta phase-locking in the orbitofrontal cortex drives cocaine choice under the influence

**DOI:** 10.1038/s41598-020-64962-w

**Published:** 2020-05-15

**Authors:** Karine Guillem, Serge H. Ahmed

**Affiliations:** 1 Université de Bordeaux, Institut des Maladies Neurodégénératives, UMR 5293, 146 rue Léo-Saignat, F-33000 Bordeaux, France; 2 CNRS, Institut des Maladies Neurodégénératives, UMR 5293, 146 rue Léo-Saignat, F-33000 Bordeaux, France

**Keywords:** Neural circuits, Reward

## Abstract

Cortical theta oscillations of neuronal activity are a fundamental mechanism driving goal-directed behavior. We previously identified in the rat orbitofrontal cortex (OFC) a neuronal correlate of individual preferences between cocaine use and an alternative nondrug reward (i.e. saccharin). Whether theta oscillations are also associated with choice behavior between a drug and a nondrug reward remains unknown. Here we investigated the temporal structure between single unit activity and theta band oscillations (4–12 Hz) in the OFC of rats choosing between cocaine and saccharin. First, we found that the relative amplitude of theta oscillations is associated with subjective value and preference between two rewards. Second, OFC phase-locked neurons fired on opposite phase of the theta oscillation during saccharin and cocaine rewards, suggesting the existence of two separable neuronal assemblies. Finally, the pharmacological influence of cocaine at the moment of choice altered both theta band power and theta phase-locking in the OFC. That is, this drug influence shifted spike-phase relative to theta cycle and decreased the synchronization of OFC neurons relative to the theta oscillation. Overall, this study indicates that the reorganization of theta phase-locking under the influence of cocaine biases OFC neuronal assemblies in favor of cocaine choice and at the expense of a normally preferred alternative, a neuronal change that may contribute to drug preference in cocaine addiction.

## Introduction

The orbitofrontal cortex (OFC) plays a crucial role in processing reward information about competing options to influence choice and preference in humans, monkeys, and rats^1–3^. OFC neurons encode incentive value and the relative preference when choosing between two rewards of the same kind (e.g., two different amounts of food)^4–10^. We recently extended these findings to choice between drugs of abuse (i.e., cocaine) and nondrug alternative rewards (i.e., water sweetened with saccharin)^11,12^. Specifically, we found that drug and nondrug rewards are selectively encoded by two non-overlapping populations of OFC neurons, and that OFC spiking activity reflect the relative preference between the two types of options^11,12^.

In addition to single cell coding, oscillatory activity of the local field potential (LFP), which results from the sum of incoming currents across all excitable membranes within a local volume^13^, also plays a fundamental role in cognitive functions and behavior^14–16^. In particular, theta oscillations (4–12 Hz) have been observed across several cortical brain regions and implicated in cognitive functions such as reward processing and goal-directed behavior^17–19^. Moreover, within individual brain areas, oscillations can synchronize neurons thereby creating coherent cell assemblies^20^. Consistent with this, it has been shown that reward information encoded by OFC neurons in rats becomes highly synchronized to OFC theta oscillation in anticipation of the reward^18^. Importantly, spike-field phase locking synchronization was stronger when anticipating a positive rather than a negative outcome, suggesting that OFC theta oscillations encode subjective reward value.

Whether OFC theta oscillations are modulated and synchronize OFC firing neuronal activity during choice between cocaine and a nondrug reward remains to be investigated. Moreover, we also sought to determine whether and to what extent the drug influence at the moment of choice could alter these OFC oscillatory activity dynamics. We recently demonstrated that while rats prefer the nondrug option when not under the drug influence, they shift their choice toward cocaine nearly exclusively when making their choice under the drug influence^21,22^. To address these questions, we analyzed the LFP data from a previous set of experiments^11^. Briefly, LFP and neuronal spiking activities were recorded in the OFC of rats trained to choose between sweet water and cocaine. Specifically, we first examined the temporal modulation of theta oscillations in the OFC and then the relationships between OFC single unit activity and theta oscillations during both sampling and choice trials. Finally, we compared OFC theta oscillatory activity and spike field phase-looking when choices were made drug free versus under the influence of cocaine.

## Results

**Figure 1:**
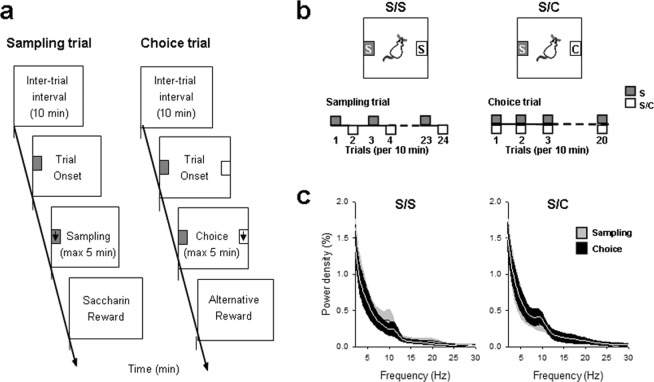
Experimental design and specific choice procedures. (**a**) Schematic description of a sampling trial and a choice trial. Rectangles represent different successive task events within each type of trial (i.e., inter-trial interval; trial onset; sampling or choice; reward delivery). (**b**) Specific discrete-trials choice procedures used in different experiments. S/S: choice between two concentrations of sweet water (20-s access to 0.2% vs 0.04% saccharin); and S/C: choice between sweet water (0.2% saccharin) and cocaine (0.25 mg, i.v). In all cases, the discrete-trials choice procedure was divided into 2 successive phases, first sampling trials (24 trials) followed by choice trials (20 trials). (**c**) Mean (± s.e.m.) LFP power spectrum density across animals during sampling (grey) and choice (black) trials in S/S and S/C groups.

In our original study^11^, we reported OFC neuronal spiking activity in rats choosing between a drug (i.e., cocaine) and a nondrug reward (sweet water). Here we looked more specifically at LFP activity in the OFC. Briefly, two groups of animals were trained to sample and choose between two levers associated with different rewards (see Behavioral Procedures; Fig. 1): between two concentrations of sweet water (S/S, n = 6), or between sweet water and cocaine (S/C, n = 5). LFP and neuronal spiking activities were recorded after stabilization of preference during a single testing session consisted of a succession of 44 discrete trials, spaced 10 min apart, and distributed into two main periods, sampling (24 trials) followed by choice (20 trials; Fig. 1b). Importantly, in the current data set, all animals strongly preferred 0.2% saccharin over any alternative outcomes, including 0.04% saccharin (89 ± 3.0% of 0.2% choices) or cocaine (92 ± 4.6% of 0.2% choices) (data not shown). We recorded the LFP and neuronal spiking in the OFC of those two groups of animals. Spectral analysis of LFP revealed one peak within the theta frequency band (4-12 Hz), with a mean of 7.9 ± 0.5 and 9.1 ± 0.5 during sampling and choice trials in the S/S group and 8.4 ± 0.7 and 9.2 ± 0.6 in the S/C group. The frequency spectrum was also characterized by the presence of lower delta oscillatory frequencies, but with no particular preferred frequency in that range.

**Figure 2:**
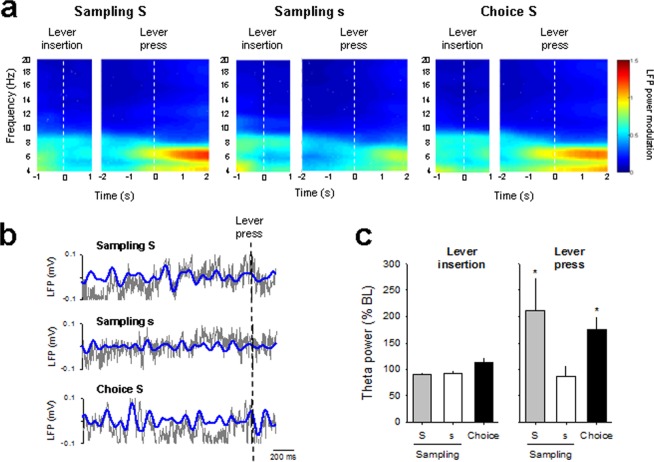
Temporal modulation of theta oscillations during choice between two concentrations of the same nondrug reward. (**a**) The mean power spectrogram of all animals during sampling and choice trials is centered around lever insertion (1 s before and 1 s after lever insertion) and lever press (2 s before and 1 s after lever press), indicated by dashed white bars. S: 0.02% saccharin and s: 0.04% saccharin. (**b**) Representative raw (grey) and theta filtered (blue) LFP trace before lever press during sampling and choice trials. (**c**) Averaged theta power (% of baseline ± s.e.m.) during sampling and choice trials for lever insertion (1 s after lever insertion) and lever press (2 s before lever press) was normalized to the average spectral power during the baseline period (a 1 s window beginning 2 s before lever insertion). Bars represented the averaged theta power for the preferred 0.2% saccharin (S, grey bars) or the alternative option (s: 0.04% saccharin, whites bars) during sampling and during choice (black bars). *p < 0.05, different from 0.04% saccharin.

We first examined the temporal modulation of theta oscillations in the S/S group by calculating mean averaged peri-event spectrograms around lever insertion (1 s before and 1 s after lever insertion) and lever press (2 s before and 1 s after lever press) during both sampling and choice trials (Fig. 2a,b). Theta power slightly decreased after lever insertion (time effect: F_(20,300)_ = 6.84; *p* < 0.001) but this effect was not significantly different between the two samplings and choice trials (time x trial type interaction: F_(40,300)_ = 1.14; NS). In contrast, theta power increased within 1 s before lever press (time effect: F_(30,450)_ = 19.36; *p* < 0.001). Notably, this increase in theta power before the press differ depending on the trials type (time x trial type interaction: F_(60,450)_ = 3.21, *p* < 0.001). To further quantify and compare theta modulation across trials type, theta power (1 s after lever insertion and 2 s before lever press) was normalized as the percentage of power during baseline (a 1 s window beginning 2 s before lever insertion, see Materials and Methods; Fig. 2c). Theta power was effectively modulated by lever press (trial effect: F_(2,10)_ = 5.40, *p* < 0.05), but not by lever insertion (trial effect: F_(2,10)_ = 2.13), indicating a predominant role of the theta oscillation during lever press. Importantly, the increase in theta power was greater for the preferred (i.e., rewarded by 0.2% saccharin) than for the non-preferred alternative option (i.e., rewarded by 0.04% saccharin) (S *vs* s, *p* < 0.05). Thus, as with OFC single activity neurons, the amplitude of theta oscillations seems to represent individual preferences. However, the theta modulation during sampling for the preferred reward was similar to that during the choice trials (S *vs* choice, NS), suggesting a similar implication of theta oscillations in the OFC during sampling and choice trials.

**Figure 3:**
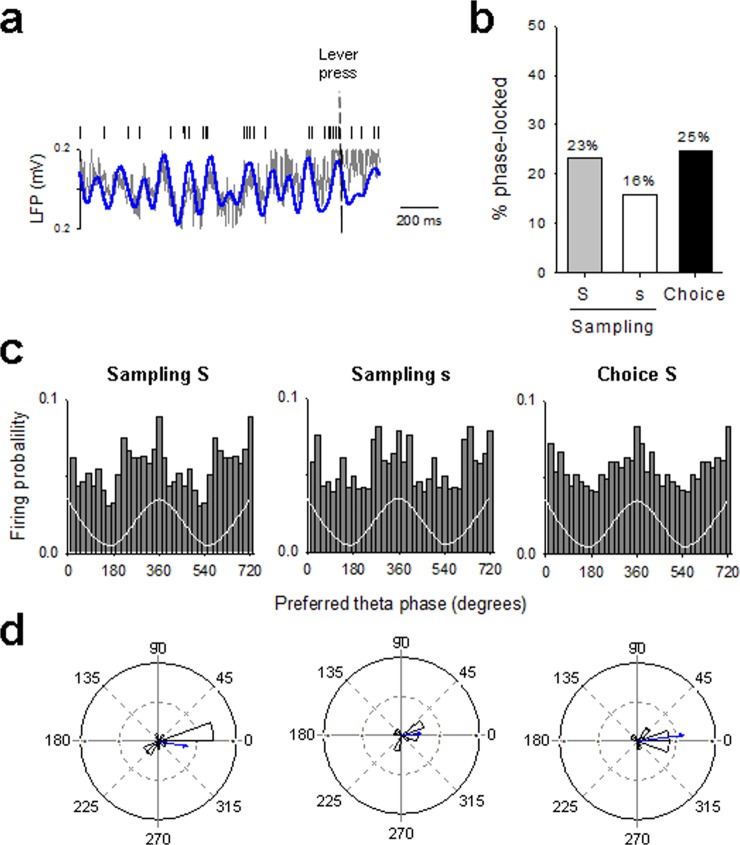
Spike-LFP phase locking during choice between two concentrations of the same nondrug reward. (**a**) Representative raw (grey) and theta filtered (blue) LFP trace and simultaneously recorded OFC single unit action potentials (top) before saccharin S sampling. (**b**) Percentage of phase-locked neurons on the theta oscillation during sampling and choice trials (2 s before lever press). S: 0.02% saccharin and s: 0.04% saccharin. Numbers indicate percentages of significantly phase-locked. (**c**) Population discharge probability and (**d**) polar histograms distribution of preferred theta phases for significantly phased-locked OFC neurons during sampling and choice trials (2 s before lever press). The white trace is a schematic of the theta cycle. Two theta circle are shown to facilitate visual comparison. Distribution of phases deviated significantly from uniformity for sampling S and choice S but not for sampling s (Rayleigh’s test *p* < 0.05, *p* < 0.01 and NS, respectively).

We next assessed whether this prominent theta band activity around lever press was related to OFC neurons spiking activity. To this end, we examined whether single OFC neurons showed consistent phase timing of action potentials relative to LFPs across sampling and choice trials (Fig. 3). It is important to note that theta power increased both before and after lever press, two effects that have been related to the anticipation and the consumption of the reward, respectively^18^. However, in this report, we based our phase-locking analysis only on the pre-press period (2 s before lever press) mainly for two reasons: 1) it was not possible to define a similar post-press consumption period in the S/C group and thus to compare the different groups; 2) OFC neurons increased their firing during the pre-press period^11^. The proportion of theta phase-locked neurons was slightly higher during sampling for the preferred reward and during choice than during sampling for the non-preferred reward (Fig. 3a,b). In all trials, these phase-locked neurons fired preferentially during the ascending phase of the oscillation (Fig. 3c) and clustered on or just prior to the peak of the theta oscillation (mean phase = 350°, 7° and 8° for sampling S, sampling s and choice, respectively; Fig. 3d). However, the distribution of the theta phases at which OFC neurons preferentially fired deviated significantly from uniform distribution during sampling for the preferred reward, but not during sampling for the non-preferred reward option (Rayleigh’s test: *p* < 0.05 and NS, for sampling S and sampling s, respectively). Importantly, the distribution of theta phases became even more concentrated during choice trials (Rayleigh’s test: *p* < 0.01), suggesting an increase in theta-mediated synchrony among OFC neurons during choice. Consistent with this, the strength of phase locking was higher during choice than during each of the two samplings (vector length = 0.38, 0.26 and 0.59 for sampling S, sampling s and choice, respectively; Fig. 3d).

**Figure 4:**
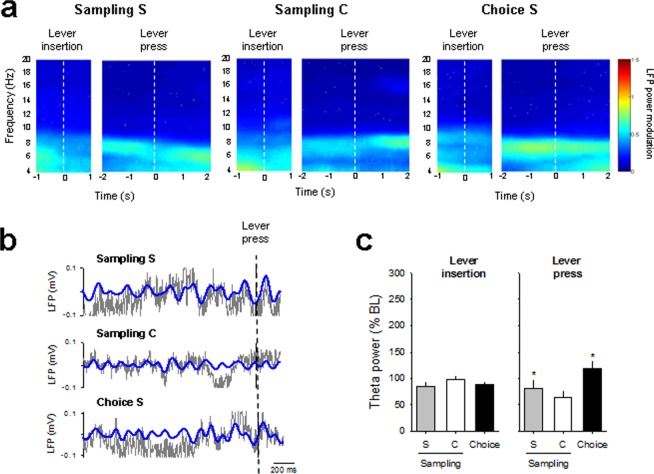
Temporal modulation of theta oscillations during choice between cocaine and a nondrug alternative. (**a**) The mean power spectrogram of all animals during sampling and choice trials is centered around lever insertion (1 s before and 1 s after lever insertion) and lever press (2 s before and 1 s after lever press), indicated by dashed white bars. S: 0.02% saccharin and C: cocaine. (**b**) Representative raw (grey) and theta filtered (blue) LFP trace before lever press during sampling and choice trials. (**c**) Averaged theta power (% of baseline ± s.e.m.) during sampling and choice trials for lever insertion (1 s after lever insertion) and lever press (2 s before lever press) was normalized to the average spectral power during the baseline period (a 1 s window beginning 2 s before lever insertion). Bars represented the averaged theta power for the preferred 0.2% saccharin (S, grey bars) or the cocaine option (C, whites bars) during sampling and during choice (black bars). *p < 0.05, different from cocaine.

**Figure 5:**
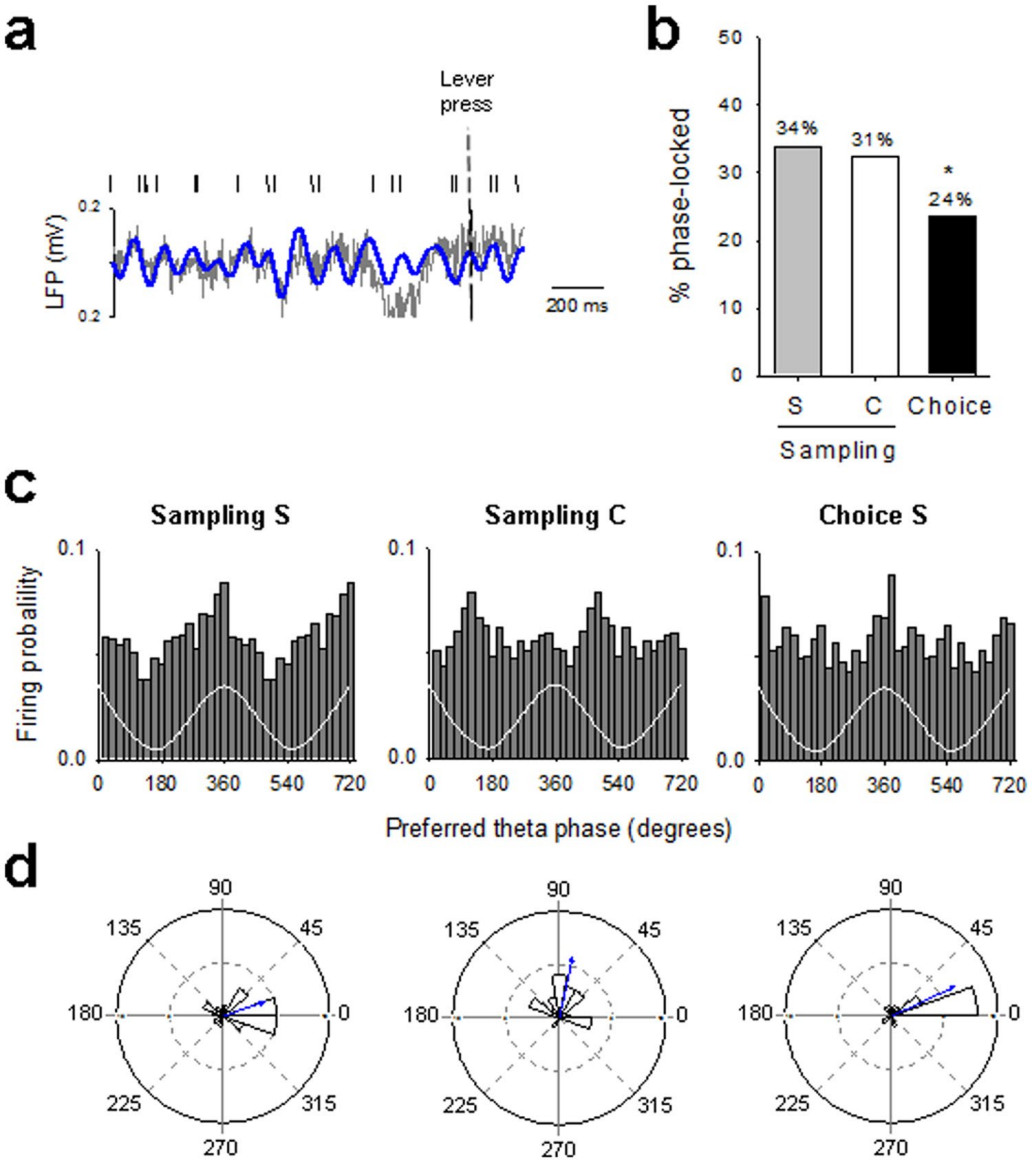
Spike-LFP phase locking during choice between cocaine and a nondrug alternative. (**a**) Representative raw (grey) and theta filtered (blue) LFP trace and simultaneously recorded OFC single unit action potentials (top) before cocaine sampling. (**b**) Percentage of phase-locked neurons on the theta oscillation during sampling and choice trials (2 s before lever press). S: 0.02% saccharin and C: cocaine. Numbers indicate percentages of significantly phase-locked neurons. *p < 0.05, different from sampling S. (**c**) Population discharge probability and (**d**) polar histograms distribution of preferred theta phases for significantly phased-locked OFC neurons during sampling and choice trials (2 s before lever press). The white trace is a schematic of the theta cycle. Two theta circle are shown to facilitate visual comparison. Distribution of phases deviated significantly from uniformity for all sampling and choice trials (Rayleigh’s test *p* < 0.05, *p* < 0.01 and *p* < 0.05 for sampling S, sampling C and choice S, respectively).

Temporal modulation of theta oscillations around lever insertion (1 s before and 1 s after lever insertion) and lever press (2 s before and 1 s after lever press) was also observed in the S/C group (Fig. 4a,b). Theta power slightly decreased after lever insertion in both samplings and choice trials (time effect: F_(20,300)_ = 4.05, *p* < 0.001; time x trial type interaction: F_(40,300)_ = 1.01, NS), but did not change significantly before lever press (time effect: F_(30,360)_ = 0.78; NS), suggesting that the presence of the drug option abolished theta power increase before lever press. Alternatively, it is also possible that different electrode placements between the S/S and the S/C groups could explain this lack of increase in theta power. However, normalized theta power 1 s before the press was still greater during choice and during sampling for the preferred than for the non-preferred alternative option (S v*s* C, *p* < 0.5 and C *vs* choice, *p* < 0.05) (Fig. 4c). Spike-LFPs phase-locking (measured during a 2 s time period before lever press) was also altered in the S/C group compared to the S/S group (Fig. 5). For instance, though the proportion of theta phase-locked neurons was similar between the two sampling trials (Fig. 5b), phase-locked neurons fired on opposite phase of the oscillation (Fig. 5c,d). Specifically, the firing probability was maximal during the ascending phase of the oscillation for saccharin sampling but on the descending phase for cocaine sampling (Fig. 5c). The mean preferred phases of significantly phase-locked neurons thus clustered on the peak of the theta oscillation for saccharin sampling but closer to the trough for cocaine sampling (Rayleigh’s test: *p* < 0.05 and *p* < 0.001 and mean phase = 20 and 78° for sampling S and sampling C, respectively; Fig. 5d), indicating the existence of two separable sub-regional clusters of neurons locking at different phases of the theta cycle. The proportion of phase-locked cells was also higher during the two sampling than during choice trials (*p* < 0.05; Fig. 5b). However, because the percentage of phase-locked cells during choice trials in the S/C and the S/S groups were similar, this difference in the proportion between sampling and choice trials seems to represent an increase in spike-LFPs phase-locking during sampling trials in the S/C group rather than a decrease during choice trials. Consistent with the saccharin sampling results, neurons fired preferentially during the ascending part of the oscillation and clustered on the peak of the theta oscillation during saccharin choice trials (Rayleigh’s test: *p* < 0.001 and mean phase = 25 °; Fig. 5c,d). The strength of phase locking was higher during choice than during each of the two samplings (vector length = 0.42, 0.59 and 0.66 for sampling S, sampling C and choice, respectively).

**Figure 6:**
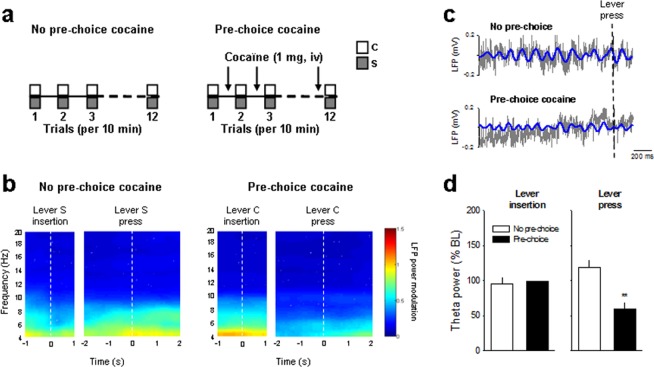
Temporal modulation of theta oscillations during pre-choice cocaine induced reversal of choice to cocaine. (**a**) Schematic representation of choice between cocaine and a nondrug alternative in 2 different conditions: without (first 12 choice trials, no pre-choice) or with pre-choice, noncontingent administration of cocaine (1 mg, i.v.) (last 12 choice trials). Each pre-choice dose of cocaine was administered 5 min before onset of choice trials. (**b**) The mean power spectrogram of all animals during choice trials without (no pre-choice cocaine) and with pre-choice cocaine is centered around lever insertion (1 s before and 1 s after lever insertion) and lever press (2 s before and 1 s after lever press), indicated by dashed white bars. S: 0.02% saccharin and C: cocaine. (**c**) Representative raw (grey) and theta filtered (blue) LFP trace before lever press without and with pre-choice cocaine. (**d**) Averaged theta power (% of baseline ± s.e.m.) during choice trials without (white bars; 2 s before lever press) and with pre-choice cocaine (black bars; 2 s before lever press) was normalized to the average spectral power during the baseline period (a 1 s window beginning 2 s before lever insertion). **p < 0.01, different from no pre-choice cocaine condition.

**Figure 7:**
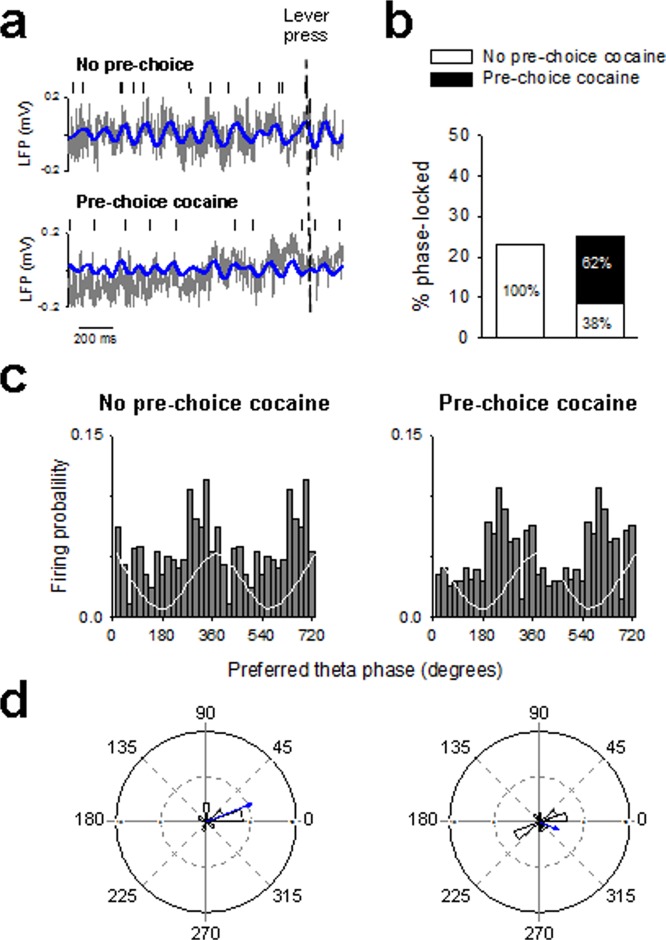
Spike-LFP phase locking during pre-choice cocaine induced reversal of choice to cocaine. (**a**) Representative raw (grey) and theta filtered (blue) LFP trace and simultaneously recorded OFC single unit action potentials (top) during choice trials without (no pre-choice cocaine) and with pre-choice cocaine. (**b**) Percentage of phase-locked neurons on the theta oscillation choice trials (2 s before lever press) without (white bars) and with pre-choice cocaine (black bars). Numbers indicate percentages of significantly phase-locked neurons. (**c**) Population discharge probability and (**d**) polar histograms distribution of preferred theta phases for significantly phased-locked OFC neurons during choice trials (2 s before lever press) without (no pre-choice cocaine) and with pre-choice cocaine. The white trace is a schematic of the theta cycle. Two theta circle are shown to facilitate visual comparison. Distribution of phases deviated significantly from uniformity for the no pre-choice but not for the pre-choice cocaine condition (Rayleigh’s test: *p* < 0.01 and NS for no pre-choice and pre-choice cocaine, respectively).

Finally, we assessed LFP activity and synchrony in a separate group of rats (n = 5) that made choices under the influence of cocaine versus not under this influence (see Behavioral Procedures). The influence of cocaine was induced by passively administering cocaine before each choice trial (see Materials and Methods; Fig. 6a), a pharmacological intervention known to shift choice from sweet water to cocaine^21,22^. As expected, in all initially saccharin preferring animals pre-choice cocaine shifted preference from saccharin to cocaine almost exclusively (from 16.6 ± 10.2% to 91.6 ± 5.2% of cocaine choices; F_(1,4)_ = 23.8; *p* < 0.01). We hypothesized that passive, pre-choice administration of cocaine will alter the theta power and the timing of neuronal firing with respect to local theta activity. Compared with the control condition where no pre-choice cocaine was administered, passive, pre-choice administration of cocaine blocked theta power increase before lever press (Fig. 6b,c) and caused a decrease in normalized theta power (normalized to a 1-s baseline window beginning 2 s before lever insertion) (t_(3)_ = 5.2; *p* < 0.01; Fig. 6d). Though the global proportion of theta phase-locked neurons was similar before and after cocaine priming (Fig. 7a,b), most of the neurons (62%) after the cocaine priming were new phase-locked ones, while only 38% of the initially phase-locked during control no pre-choice cocaine condition remained phase-locked after the priming. Moreover, pre-choice administration of cocaine shifted spike phase relative to theta cycle and caused OFC neurons to fire at earlier phases relative to the theta oscillation (Fig. 7c,d). Notably, OFC neurons changed their theta phase-locking, shifting from a preferred 22° phase to a 337° phase after pre-choice cocaine administration. Importantly, this shift in spike phase was associated with a decreased synchronisation as the theta phase occurrence became uniformly distributed after pre-choice cocaine administration (Rayleigh’s test: *p* < 0.01 and NS for no pre-choice and pre-choice cocaine, respectively; Fig. 7d).

## Discussion

Cortical theta oscillations of neuronal activity is a fundamental mechanism driving cognitive functions and behavior^14,19^. Here, we examined OFC theta oscillation and spike-field phase-locking in rats trained to sample and choose between cocaine and a preferred nondrug alternative. We found that OFC theta oscillations were associated with subjective value and choice preferences. In both the S/S and the S/C groups, normalized theta band power was higher during sampling for the preferred reward and during choice than during sampling for the non-preferred reward. Though significant, the increase in theta power in the S/C group was lower than that in the S/S group, an effect that could be due to the presence of the drug as an option in the S/C group, or to different electrode placements between the two groups. Moreover, in both groups, the strength of phase locking was higher during choice than during sampling. This locking is manifested by the fact that OFC neurons consistently fired at a particular narrow range of phases on the theta oscillation. However, some differences exist. During sampling between two different concentrations of saccharin, OFC neurons fired on the same phase of the theta cycle: near the peak of the theta oscillation for both samplings. In contrast, OFC phase-locked neurons fired on opposite phase of the oscillation during saccharin and cocaine samplings: near the peak of the theta oscillation for saccharin sampling but near the trough for cocaine sampling. Brain oscillations have been shown to provide volleys of inhibitory inputs onto pyramidal cells, thereby providing windows of alternating reduced and enhanced excitability of pyramidal cells in a temporally coordinated manner. This would allow segregation of excitatory cells into functional groups, often referred as to as neuronal assemblies^23,24^. Together, these results thus suggest that two separable neuronal assemblies are recruited during saccharin and cocaine sampling. This idea is also consistent with our previous OFC single activity analysis showing that two non-overlapping populations of OFC neurons encoded cocaine and saccharin reward samplings^11^.

During choice trials, the distribution of theta phases as well as the strength of theta phase-locking became even more concentrated than during sampling trials, indicating an increase in theta-mediated synchrony among OFC neurons during choice. This finding is consistent with previous studies on spatial working memory tasks showing a higher theta phase-locking in the mPFC during choice versus spatial sampling^25–27^. Synchronization of action potential firing through spike-LFP phase-locking has been proposed to promote the relay of relevant information and drive firing in the proper targets with higher probability^28–30^, an effect that could directly contribute to the preferential processing of task-relevant stimuli in downstream areas^31^. The enhanced strength of theta band phase-locking during choice trials may thus potentiate the efficiency with which OFC output affects target brain regions involved in action selection^32,33^ to facilitate choice and preferences. Consistent with this, theta oscillations have been shown to facilitate plasticity^34,35^. Whether this increased theta phase-locking in the OFC leads indeed to changes in synchrony with other brain structures remains to be investigated.

In addition to theta rhythm, OFC field potentials were also characterized by increased gamma power and gamma phase-locking neurons during an olfactory discrimination task^36^. This gamma phase-locking is learning-dependent, but not stimulus-selective, and is expressed by a group of neurons whose firing rate is inversely related to the strength of gamma synchronization, suggesting that OFC gamma-band synchronization reflects behavioral inhibitory control. It will be interesting in future research to further assess the role of gamma oscillations and gamma phase-locking OFC neurons in choice behavior between a drug and a nondrug reward.

We also found that non-contingent cocaine administration (i.e., 1 mg) before each choice trial, a pharmacological intervention known to shift choice to cocaine^21,22^, alters both theta band power and theta phase-locking in the OFC. First, and perhaps surprisingly, this shift in preference from saccharin to cocaine was associated with a strong decrease, rather than an increase, in normalized theta power, a finding consistent with previous studies^37–39^. However, it is important to note here that contrary to the S/S and the S/C groups which expressed their normal preference, animals under the influence of cocaine at the time of choice expressed a preference that was opposite to their normal preference. Our hypothesis is that the decrease in theta power induced by cocaine at the time of choice may reflect a drug-induced neuronal suppression of the normally preferred saccharin option, thereby favouring by default the choice of the drug option. Second, pre-choice cocaine administration shifted spike phase relative to theta cycle and caused OFC neurons to fire at earlier phases relative to the theta oscillation. Modification of preferred phase relative to theta oscillations has been previously shown in the mPFC during learning and experience changes in behavior^27,40^. Similarly, spike phase shifts have been observed following dopamine or serotonin applications^27,41,42^, an effect that could contribute to the present shift in theta phase after cocaine pre-trial administration. Importantly this cocaine-induced shift in theta phase-locking in the OFC was associated with a decreased synchronisation and, at the behavioral level, to a profound shift in preference towards cocaine. As mentioned above, synchronization of pyramidal neurons activity within individual brain areas is supposed to increase information transfer to target brain regions through synaptic plasticity processes. The change in phase preference and the desynchronization of OFC neurons firing activity after cocaine pre-trial administration may thus profoundly disrupt neural network assemblies and modify their impact on postsynaptic target areas.

GABAergic inhibitory interneurons have been shown to play a crucial role in the generation of theta cycle in several brain areas including the cortex^43–45^. Indeed, interneurons can modulate theta oscillations and control dynamically the firing phase of pyramidal neurons^46,47^. It is therefore possible that the shift in theta phase after pre-choice cocaine was due to a change in interneurons’ firing activity. Consistent with this hypothesis, a preliminary analysis revealed that the activity of OFC interneurons, as identified by their electrophysiological profile^48^, increased dramatically and rapidly after passive pre-choice administration of cocaine (*F*_(140, 980)_ = 1.8; *p* < 0.001, K. Guillem et al., unpublished data) and remained significantly elevated during several minutes. Such an increase in inhibitory efficacy after cocaine pre-trial administration could constrain the pyramidal cells to fire primarily at an earlier phase (i.e., the trough) of the theta cycle, hence decreasing synchrony. Future research will need to confirm this hypothesis.

Regardless of the mechanism involved, this shift in phase-locking could cause a profound reorganization of the dynamics of OFC network leading to the formation of different neuronal assemblies associated with different behavior. Consistent with this, distinct ensembles of phase-locked neurons were recruited under control condition and after cocaine priming. Under cocaine priming, only a minority (31%) of phase-locked neurons were also phase-locked under control condition while the vast majority (69%) was not. Importantly, these two phase-locking ensembles are associated with two different choice behaviors: one phase-locking ensemble fired close to the peak of the theta oscillation and is associated with saccharin choices, while the other phase-locking ensemble fired closer to the trough and is associated with cocaine choices. Thus, our results suggest that pre-choice cocaine decreased the activity of the neuronal assembly associated with saccharin choices, while it increased the activity of another neuronal assembly associated with cocaine choices. Though the mechanisms underlying this shift in phase-locking remain to be fully elucidated, this aberrant synchronization within the OFC and consequent of the neurocircuitry underlying choice may contribute to drug preference in cocaine addiction.

## Materials and Methods

### Animal and surgeries

Male Wistar rats (n = 16) were surgically prepared with a catheter in the right jugular vein and rectangular arrays of 16 teflon-coated stainless steel microwires (2 rows of 8 wires separated from each other by 0.25 mm; MicroProbes Inc, Gaithersburg, MD) were implanted unilaterally in the OFC [AP: + 2.5 to +3.7 mm, ML: 1.5 to 4.5 mm, and DV: −5.0 mm relative to skull level] as previously described in detail elsewhere^11,48^. Rats were initially housed two per cage. After surgery, they were housed individually in a temperature- and ventilation-controlled environment under a reversed 12 h light/dark cycle (for additional details, see^11^). All experiments were carried out in accordance with standard ethical guidelines (European Communities Council Directive 86/609/EEC) and approved by the committee on Animal Health and Care of Institut National de la Santé et de la Recherche (Agreement A5012052).

### Experimental stages and procedures

#### Behavioral apparatus

Operant chambers (30 × 40 × 36 cm) used for all behavioral training and testing (Imetronic, Pessac, France) have been described in detail elsewhere^11,46^. Briefly, each chamber was equipped with 2 automatically retractable levers (Imetronic), a commercially-available lickometer circuit (Imetronic), two syringe pumps, a single-channel liquid swivel (Lomir biomedical Inc., Quebec, Canada), an electrical commutator (Crist Instrument Inc., Hagerstown, MD), and two pairs of infrared beams to measure horizontal cage crossings.

#### Discrete-trials choice procedure

Animals were first trained on alternate days to respond on both levers under a fixed-ratio 1 (FR1 time-out 20 s) schedule to obtain different rewards depending on the group: between two concentrations of sweet water (S/S, n = 6), or between sweet water and cocaine (S/C, n = 5) (Fig. 1a,b). In the S/S group, one rat did not have good LFPs recordings and was excluded from this analysis, leaving 6 animals in the group. In the S/C group, all the 5 nondrug-preferring rats had good LFPs recording and were thus analyzed in the present study. In the S/S group, one lever was associated with 20-s access to water sweetened with 0.2% saccharin, and the other with 20-s access to water sweetened 0.04% saccharin. In the S/C group one lever was associated with water sweetened with 0.2% saccharin and the other with an intravenous infusion of cocaine (0.25-mg in 5 s). Rats were next trained under a discrete-trials choice procedure until stabilization of preference, as previously described in detail elsewhere^11^. Briefly, each session consisted of 44 trials spaced 10 min apart and divided into two successive phases: sampling (24 sampling trials) and choice (20 choice trials) (Fig. 1b). During sampling, the two levers were presented separately on alternate trials and rats have to within 5 min on the presented lever to receive the associated reward. During choice, the two levers were presented simultaneously on the same trials and rats were free to choose between these two levers to receive the corresponding reward. If rats failed to respond on either lever within 5 min, both levers retracted and no reward was delivered.

#### Non-contingent cocaine administration before choice

We recently found that a non-contingent administration of cocaine (i.e., 1 mg, i.v.) before each choice trial caused rats to immediately shift their preference from sweet water to cocaine^21^. A group of rats (*n* = 5) was first trained to choose between cocaine and saccharin until stabilization of their choice performance before the non-contingent pre-trial administration of cocaine test, as described elsewhere^11^. This was done in one session using a choice procedure identical to that described above during a total of 24 choice trials, except that the last 12 choice trials were each preceded 5 minutes before by a pre-choice administration of cocaine (i.e., 1 mg, i.v.).

### *In vivo* electrophysiological recordings

#### Recording and spike sorting

During recording sessions, electrodes will be connected to headstages and voltage signals from each electrode will be recorded, amplified, bandpass filtered (from 250 Hz to 8 kHz for unit activity and from 0.7 Hz to 170 Hz for LFPs) and digitally captured (Plexon Inc, Dallas, USA). Neuronal activity was digitized at a rate of 40 kHz and LFPs were down sampled to 1 kHz. Behavioral events in the operant task will be streamed to the Plexon system via TTL pulses to allow the neuronal data to be accurately synchronized to these behavioral events. Single units spike sorting was performed off-line using Offline Sorter software (Plexon) and analyzed using Neuroexplorer (Nex Technologies) as previously described^11,12^. The quality of individual-neuron recordings will be ensured with the following criteria: <3% of all interspike intervals exhibited by the unit are <2000 µs, and the average amplitude of the unit waveform is at least three times larger than that of the noise band (>3:1 signal-to-noise). These units were classified into putative interneurons and pyramidal neurons according to the waveform spike width and average firing rate, as previously described^48^. Electrophysiological data were analyzed using NeuroExplorer (Plexon) and Matlab (Mathworks, Natick, MA).

#### Spectral analysis

The power spectral density (PSD) of each LFP was calculated with the NeuroExplorer PSD function using a multitaper fast Fourier transform (FFT), with a Hamming window of 2048 points (2 s), 50% overlap and 1 Hz resolution. PSD values were expressed as the percent of the total power spectrum within the frequency range considered (1–30 Hz). For each trial of a given condition (i.e., sampling or choice trials), the power density analysis was performed using a 2-s time window period centered around the behavioral events of interest (i.e., lever insertion or lever press) and averaged across each trial of a given condition (i.e., sampling or choice trials) to obtain a mean power spectrum. To measure the temporal modulation of LFP theta oscillations during specific behavioral events of interest (i.e., lever insertion and lever press during both sampling and choice trials) peri-event spectogramms (PESPs) of power density were calculated (NeuroExplorer) and averaged across animals (Matlab), using the following time windows: 1 s before and 1 s after lever insertion, and 2 s before and 1 s after lever press. The LFP theta frequency band was taken as the frequency at maximum power within the 4–12 Hz frequency band of the time-averaged spectrogram. Averaged theta power (% of baseline ± s.e.m.) was normalized to the average spectral power during the baseline period (a 1 s window beginning 2 s before lever insertion).

#### Phase-locking analysis

To investigate the relationship between single unit activity and LFPs we performed spike-LFP phase locking for each trial of a given condition (i.e., sampling or choice trials). This phase-locking analysis was conducted only on the pre-press period (2 s before lever press) because this period was preserved and identical between the two groups, and OFC neurons increased their firing during that pre-press period^11^. LFP data were first Hann filtered to the theta (4–12 Hz) (NeuroExplorer). The phase vector was then calculated using the Hilbert transform of the filtered signal yielding values ranging from 0–360° (180° corresponds to the trough), thus providing an indication of the current phase position of the oscillation over time. Only neurons with >50 spikes during the period analyzed were used. Phase-locking was quantified as the circular concentration of the resulting phase vector distribution which is defined as the mean resultant vector length of the phase angles. The resultant vector length is a real number ranging from 0 (low phase-locking) to 1 (perfect phase-locking). Measures based on mean vector length can be biased by the number of spikes entered into the computation. Thus, we tried to prevent this bias by keeping constant the number of spikes (n = 50) used when comparing between different rewards and trials types, as was done previously^18,49^. The statistical significance of spike-LFP phase locking was addressed using the circular statistics (Oriana software, KovachComputing Services). A neurons was significantly phase-locked if the distribution of spike phase angles deviated from a uniform circular distribution (Rayleigh’s test, *p* < 0.01).
